# Teaching methods in the prevention of catheter-related bloodstream infection: a systematic review

**DOI:** 10.1186/2047-2994-4-S1-P90

**Published:** 2015-06-16

**Authors:** CES Pelaes, TR Santos, MC Padoveze

**Affiliations:** 1 Nursing School of University of Sao Paulo, Sao Paulo, Brazil

## Introduction

Catheter-related Bloodstream Infections (CRBSI) are most worrisome event due to the morbidity and mortality associated. Looking for elaboration of educational programs developed by nurses, revealed the following research question: "What are the methods and teaching techniques that have proved to be effective in reducing CRBSI rates?"

## Objectives

To identify and to describe the methods and teaching techniques applied to training programs for healthcare workers aiming the CRBSI prevention.

## Methods

A Systematic Literature Review was performed following the PICO [1] strategy between January to April of 2013, using standardized controlled and non-controlled search terms. The following databases were searched: PubMed/MEDLINE, CINAHL, LILACS, EMBASE, ERIC and Web of Science, without restriction of language and time. The articles were included if they met the quality assessment criteria [2].

## Results

Ten studies were included among 300 gathered. Five categories of **Teaching Methods** were assessed with their respective ability of use (*Expository* 100%, *Joint Development Method 80%*, *Individual Work* 60%, *Group Work* 0% *and Special Activities* 0%). Twenty-six **Teaching Techniques** were presented according to each category. The Verbal Technique (80%) and Dialogued Conversation (80%) showed to have higher affinity of use, respectively related to the Expository and Joint Development Methods. Ten **Instructional Resources** and five **Analysis Methods** were also assessed according to each related category and with its affinity of use.

## Conclusion

Different educational interventions found to be effective in reducing CRBSI. It was not possible to identify any one method as more effective. None of the studies included cited any specific theoretical instructional approach able to underlie their teaching technique during their training sessions. The infection preventionist needs to explore and achieve different teaching skills during educational training once the Education is one of the strategies for preventing Healthcare Associated Infections.

## Disclosure of interest

None declared.
